# In Situ Laser Fenestration for Subclavian Preservation During Aortic Dissection Repair

**DOI:** 10.1155/crvm/1224237

**Published:** 2026-04-28

**Authors:** Charlotte Burch, S. Ayesha Farooq, Mark Levy, Francisco Albuquerque, Robert Larson, Daniel Newton

**Affiliations:** ^1^ School of Medicine, Virginia Commonwealth University, Richmond, Virginia, USA, vcu.edu; ^2^ Division of Vascular Surgery, Department of Surgery, Virginia Commonwealth University, Richmond, Virginia, USA, vcu.edu

**Keywords:** aortic dissection, laser fenestration, TEVAR

## Abstract

Laser fenestration is a novel technique to create an opening in an endograft to maintain perfusion to the arch vessels during thoracic endovascular aortic repair (TEVAR). A 40‐year‐old female who had undergone an emergent ascending aortic repair for acute dissection presented with aneurysmal degeneration of the descending thoracic aorta. She then underwent debranching of the left carotid and innominate arteries, followed by TEVAR. Herein, we describe our approach during repair with in situ laser fenestration of the endograft to preserve subclavian arterial flow.

## 1. Introduction

Acute aortic dissection has an incidence of 3 cases per 100,000 person‐years and has a male‐to‐female preponderance of 3:1. Of note, Stanford Type A aortic dissection accounts for nearly two‐thirds (62%) of cases [1]. As such, endovascular aortic repair has become the mainstay of treatment for thoracic aortic pathology, including Type B dissections. In fact, data from two multicenter randomized controlled trials showed thoracic endovascular aortic repair (TEVAR) to be favorable over medical therapy for Type B aortic dissections with an improved overall survival and delayed disease progression [2, 3]. Approximately one‐third of patients undergoing TEVAR require coverage of the subclavian artery to achieve an adequate proximal landing zone. Therefore, the Society for Vascular Surgery Committee on Aortic Disease recommends revascularization of the subclavian artery for elective cases to prevent the risk of arm ischemia, posterior circulation stroke, or spinal cord ischemia [4]. However, endovascular revascularization of arch vessels remains a tremendous challenge. Several methods are described including extra‐anatomic bypass, parallel stent grafting, and branched endograft deployment [5]. In addition, in situ fenestration is a novel technique initially reported in 2004. It involves puncturing and dilating the graft fabric to maintain perfusion to the branch vessels [6]. Three main methods have been described including needle, radiofrequency, and, most recently, laser fenestration. Unlike other methods, laser fenestration can be used in areas of tortuosity and creates a precise 2–3 mm opening in the graft that is reproducible and maintains the integrity of the endograft [7]. This technique has been used in numerous aortic pathologies including Type A and B dissections, aneurysms, penetrating ulcers, traumatic injuries, and coarctations [8]. We present a case of TEVAR with subclavian revascularization using in situ laser fenestration for aneurysmal degeneration of the descending thoracic aorta due to chronic dissection.

## 2. Description of Case

**Figure 1:**
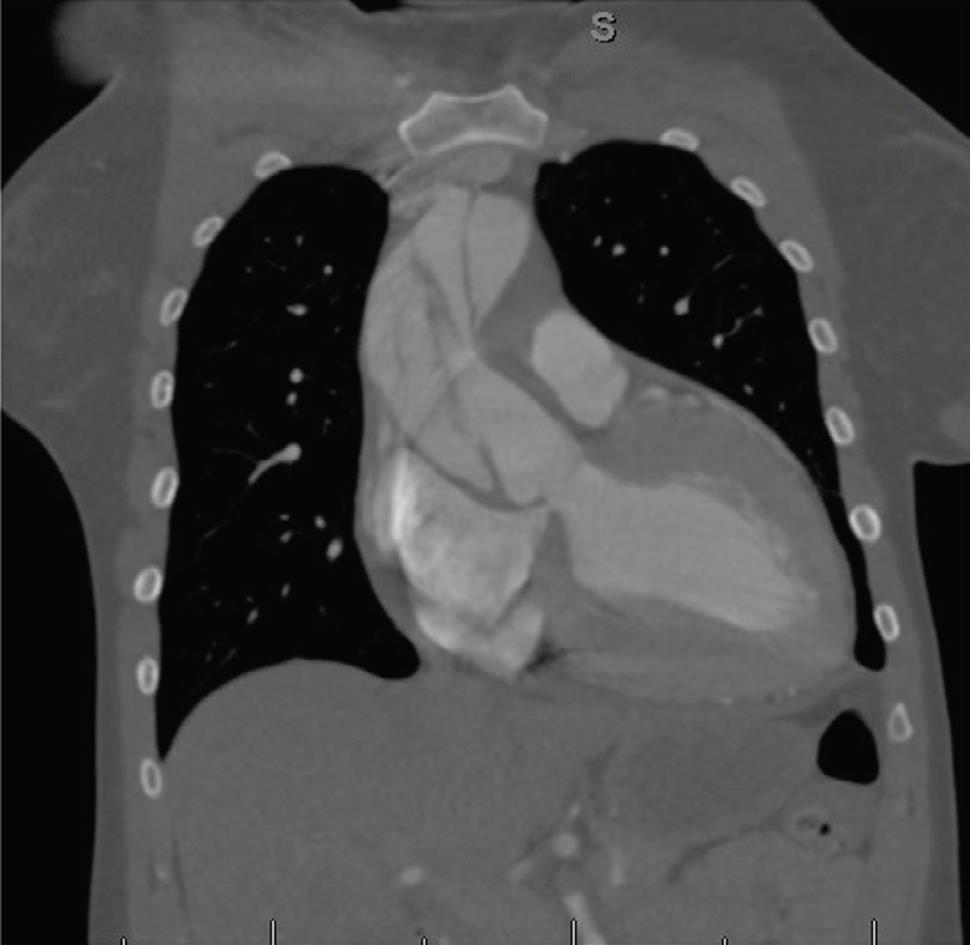
Coronal CT chest demonstrating Stanford Type A aortic dissection.

**Figure 2:**
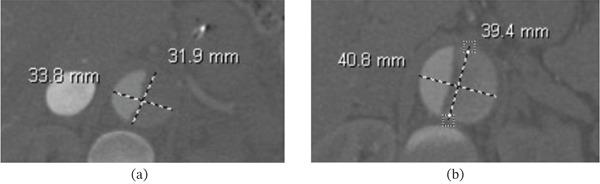
Surveillance CT scan at 1‐year follow‐up demonstrated aneurysmal degeneration of the aortic dissection from (a) 34 × 32 to (b) 41 × 39 mm.

A 40‐year‐old female presented with severe substernal, tearing chest pain radiating to her back of 5‐h duration, starting after cocaine use. In addition, she reported bilateral leg pain and numbness. Pertinent medical history included uncontrolled hypertension, heart failure with preserved ejection fraction, and chronic cocaine use. On exam, she had a blood pressure of 198/94 and a heart rate of 72 bpm. Cross‐sectional imaging demonstrated a Stanford Type A acute aortic dissection involving the ascending aorta, the aortic arch, and the descending aorta extending down to the bilateral iliac arteries (Figure 1). She underwent emergent median sternotomy, deep hemiarch replacement with a 28 mm Gelweave graft, and aortic valve resuspension with three pledgeted sutures. She developed acute renal failure postoperatively, requiring short‐course hemodialysis; however, she had renal recovery and was discharged on postop Day 11. Ten months after the initial surgery, she underwent redo sternotomy for left carotid and innominate artery debranching with 12‐10‐10 mm Gelweave graft. Surveillance CTA at 1‐year follow‐up showed approximately 1 cm aneurysmal degeneration of the residual dissection, measuring 4.1 × 3.9 from 3.3 × 3.1 cm (Figure 2). Given her past medical and surgical history and with hopes of more favorable remodeling with early intervention, we planned for TEVAR with left subclavian revascularization using in situ laser fenestration and covered stent placement.

**Figure 3:**
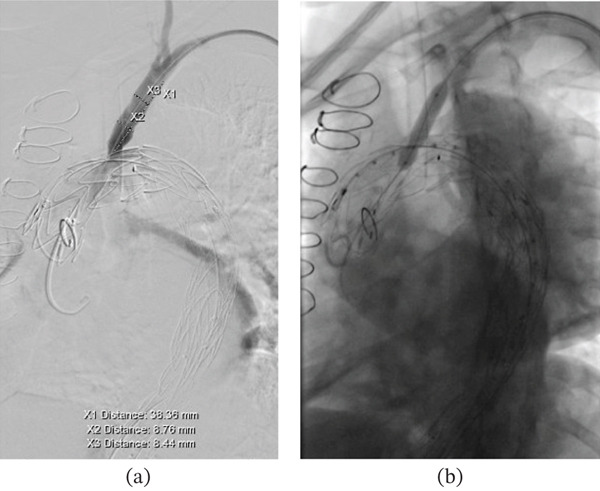
Angiography demonstrating (a) laser fenestration of the aortic stent graft at the left subclavian artery, followed by (b) balloon angioplasty of the fenestration.

The patient was brought to the hybrid OR. The procedure was performed under general anesthesia, using multimodal neuromonitoring including somatosensory and motor‐evoked potentials as well as EEG monitoring. We obtained bilateral femoral and left brachial artery percutaneous access. Following systemic heparinization, an aortogram was performed. After determining the proximal landing zone just distal to the ascending aortic bypass graft, a 36 × 200 mm Medtronic Valiant stent graft was deployed covering the left subclavian artery (Zone 0) (Figure 3a,b).

We then turned our attention to the laser fenestration part of the case. Through the left brachial artery, a wire and a vertebral catheter were advanced up to the left subclavian artery. A Rosen wire was placed, followed by a conformable sheath which was placed to about the orifice of the left subclavian, which was covered by the endograft. Through the sheath, angiography was performed to identify the landing zone since the dissection extended into the proximal left subclavian artery. A Spectranetics Turbo‐Elite 2.3‐mm laser ablation catheter was brought into position through the conformable sheath orthogonal to the graft. The laser parameters were set to a wavelength of 308 nm, 45 mJ/mm^2^, and 25 pulses/s. The laser was then activated for 3 s and advanced through the wall of the stent graft without difficulty. A 4 mm angioplasty balloon was then used to dilate the fenestration over a Rosen wire. Next, an 8 × 39 mm balloon‐expandable VIABAHN stent was deployed in the proximal left subclavian artery (Figure 3a,b).

**Figure 4:**
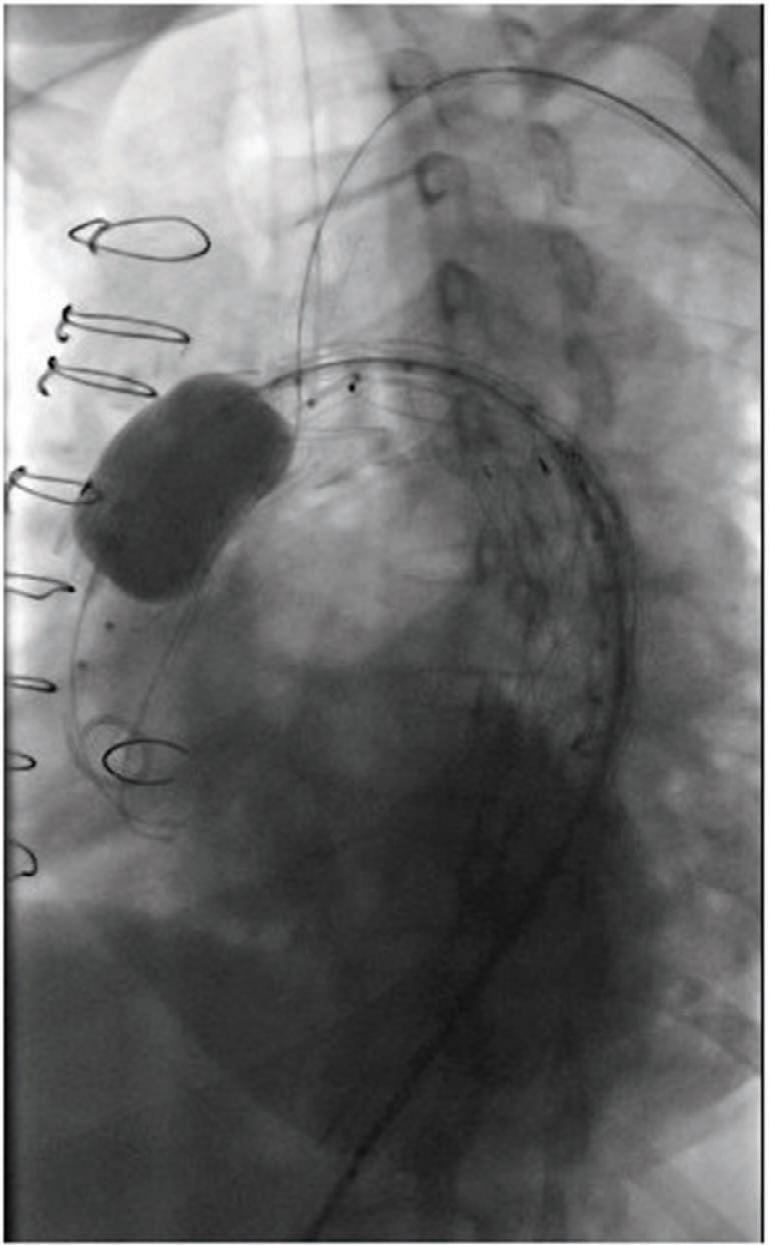
Angiography demonstrating the Reliant balloon angioplasty of the proximal end‐graft to enhance the proximal graft seal.

**Figure 5:**
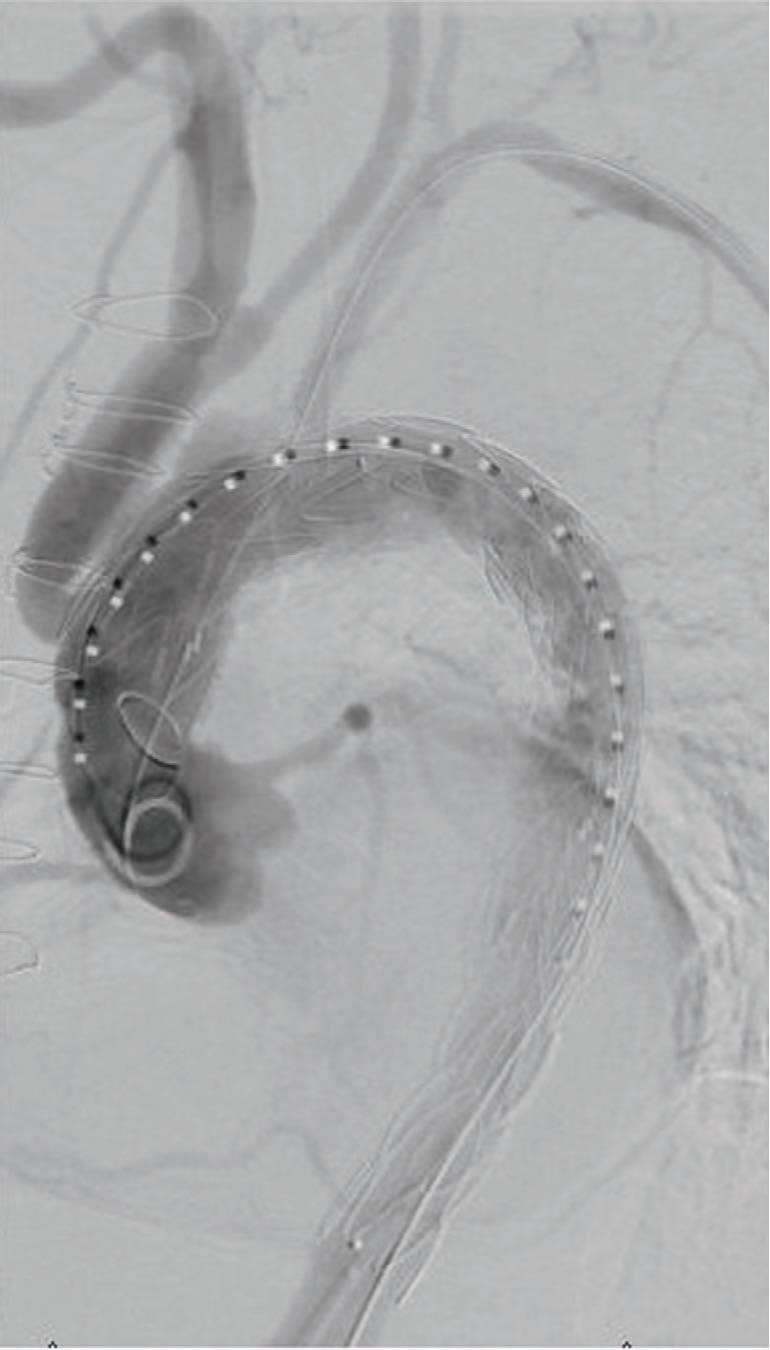
Completion angiography demonstrating TEVAR with preserved flow to the left subclavian artery as well as the debranched innominate and left common carotid arteries.

Finally, we deployed a second 40 × 150 mm Medtronic Valiant aortic stent graft in mid‐to‐distal thoracic aorta (Zone 5). Completion angiography was performed, which showed some flow around the graft into the innominate stump. Therefore, a Reliant balloon was brought into the proximal landing zone and inflated (Figure 4). We performed another angiogram which showed improvement, although there was minor flow into the innominate stump (Figure 5). There was no flow into the aneurysmal descending thoracic aorta/false lumen, no stent graft–induced new entry tears, and no retrograde dissection. Bilateral groins were closed with closure devices; then, brachial artery cutdown and arteriotomy closure were performed. The patient had palpable distal pulses and a palpable left radial pulse. She recovered in the ICU postoperatively and was discharged on postoperative Day 3. She continues to do well on 1‐year follow‐up with no evidence of endoleak on imaging.

## 3. Discussion

Aneurysmal degeneration of the thoracic aorta can develop in up to 40% of patients with Type B aortic dissections managed with medical therapy alone. Endovascular stent graft placement seals the aortic entry tear, thereby inducing false lumen thrombosis and reducing the risk of complications from Type B aortic dissection. In fact, authors of the INSTEAD‐XL trial found that in a cohort of 140 patients with uncomplicated Type B aortic dissection, TEVAR was associated with lower mortality and reduced risk of disease progression compared with medical therapy alone [9]. Similarly, Fattori et al. demonstrated that among 1192 dissection patients, one‐fourth (25.2%, *n* = 276) underwent TEVAR for dissection. Although there was no difference in short‐term outcomes, at 5‐year follow‐up, the TEVAR group had a lower mortality rate (15.5% vs. 29.0%, *p* = 0.018) [10]. An interesting aspect of the current case is the relatively young age, female sex, and cocaine use of our patient. Her history of cocaine use predisposes her to aortic pathology through acute sympathetic surges causing transient severe hypertension and increased aortic wall shear stress, which may precipitate intimal injury [11]. Her presentation at a young age suggests a nondegenerative mechanism of aortic injury, supporting the role of acute toxic exposures, such as cocaine, rather than age‐related medial degeneration alone. In this context, the International Registry of Acute Aortic Dissection (IRAD) enrolled over 3000 patients over a 15‐year period. Of note, cocaine use was implicated in 1.8% (*n* = 63) of cases and was associated with younger age groups [12].

In situ fenestration was initially described in 2004 as a means to avoid sacrificing the subclavian artery during TEVAR. In 2009, Murphy et al. reported the use of an endoluminal laser to puncture a thoracic endograft in a young trauma patient [13]. Data from the United States indicate promising midterm outcomes; however, these are limited to small single‐center series. For example, in a cohort of 22 patients undergoing urgent or emergent TEVAR, Redlinger et al. reported laser fenestration to be effective in preserving subclavian flow. At a mean follow‐up of 11 months, two (9%) patients required reintervention for Type II endoleak, and the overall mortality rate for the entire cohort was 13% (*n* = 3) [14]. In a separate analysis of 24 patients including 13 elective cases, Evans et al. noted seven (32%) patients required reinterventions to address endoleaks or stenoses [15]. Of note, a Chinese study reported the largest cohort of patients undergoing laser fenestration including 148 cases with good outcomes overall. Postoperative endoleaks occurred in seven patients with only one undergoing intervention. Interestingly, the authors also reported postimplantation retrograde aortic dissection occurring in three patients as well as air embolism causing TIA in three patients due to balloon rupture from contact with metallic endograft struts [16]. Laser fenestration can also be performed on an aberrant right subclavian artery, thereby highlighting the broad applications of this technique [15, 16]. Collectively, these data demonstrate high technical success and a complication and mortality rate lower than open repair. Additionally, laser fenestration compares favorably to parallel stent grafting with a lower endoleak rate (4.8% vs. 12.9%) [17]. Several technical considerations should be implemented to further minimize the risk of complications, including precise perpendicular apposition of the laser catheter to the graft fabric, controlled laser energy activation with immediate guidewire passage, stepwise balloon dilation, and subsequent covered stent placement [18]. Nonetheless, close surveillance is necessary to monitor for Type III endoleaks or in‐stent stenoses.

A pooled analysis of experimental studies suggested two biomechanical factors influencing the durability of laser fenestration repair: graft fabric and fenestration quality. For example, multifilament polyester grafts appear to be the most resilient compared with ePTFE or monofilament grafts. In fact, Jayet et al. reported ePTFE grafts to be highly susceptible to endoleaks due to their lack of recoil [19]. Further, Lin et al. noted ePTFE grafts to emit toxic compounds, such as hydrogen chloride and trifluoroacetate, during laser fenestration [20]. Previous authors have indicated that laser creates a clean fenestration in Dacron material while preserving graft integrity. Therefore, we opted to use a Medtronic Valiant stent graft as it is a multifilament Dacron graft.

## 4. Conclusion

In conclusion, endovascular reconstruction of the aortic arch vessels remains a considerable challenge to the vascular surgeon. Laser fenestration is a viable option for patients who are not candidates for open surgery or have anatomical constraints precluding commercially approved devices. In turn, multicenter data are needed to enhance our understanding of the applicability and determine long‐term outcomes for this technique.

## Author Contributions

All authors participated in the following: substantial contributions to the study conception and design, data acquisition or analysis, and data interpretation; drafting the manuscript or reviewing it critically for important intellectual content. Guarantor of the integrity of the entire study: D.N.; study conception and design: C.B. and S.A.F.; literature search: C.B. and S.A.F.; clinical studies: C.B. and S.A.F.; manuscript preparation: C.B. and S.A.F.; manuscript review: C.B. and S.A.F.

## Funding

No funding was received for this manuscript.

## Disclosure

All authors approved the final version of the manuscript.

## Conflicts of Interest

None of the authors have a conflict of interest to disclose.

## Data Availability

The data that support the findings of this study are available on request from the corresponding author. The data are not publicly available due to privacy or ethical restrictions.
